# F225. LEVODOPA AUGMENTATION OF ANTIPSYCHOTICS FOR THE TREATMENT OF NEGATIVE SYMPTOMS IN SCHIZOPHRENIA

**DOI:** 10.1093/schbul/sby017.756

**Published:** 2018-04-01

**Authors:** George Foussias, Naren Rao, Gagan Fervaha, Carol Borlido, Lillian Haber, Hiroyoshi Takeuchi, Romina Mizrahi, Gary Remington

**Affiliations:** 1 Centre for Addiction and Mental Health; 2 National Institute of Mental Health and Neurosciences, Bangalore; 3 Keio University, School of Medicine; 4 Centre for Addiction and Mental Health, University of Toronto

## Abstract

**Background:**

Negative symptoms (i.e., motivation deficits and diminished emotional expression) are prevalent in schizophrenia and consistently linked with functional impairment for affected individuals. Despite advances in psychopharmacology for schizophrenia, there remain no effective treatments for these negative symptoms. Older literature, however, suggests that levodopa in conjunction with antipsychotic treatment can have beneficial effects for patients with schizophrenia. While supporting the safety and potential efficacy of dopamine augmentation, these studies did not evaluate effects within specific symptom domains, particularly negative symptoms. This open-label pilot study was conducted to evaluate the preliminary efficacy and safety of levodopa augmentation of antipsychotics for the treatment of negative symptoms.

**Methods:**

Ten stable outpatients with schizophrenia between the ages of 18 and 60 were enrolled. All were treated with stable atypical antipsychotic monotherapy for at least eight weeks, and had a minimum total score of 30 on the Scale for the Assessment of Negative Symptoms (SANS). Participants were treated with adjunctive open-label levodopa/carbidopa, with dose titration to levodopa 300mg TID over 38 days as tolerated, and dose maintenance for the remainder of the eight-week trial. Baseline assessments consisted of the SANS, Scale for the Assessment of Positive Symptoms (SAPS), Brief Psychiatric Rating Scale (BPRS), Clinical Global Impression (CGI), Calgary Depression Scale for Schizophrenia (CDSS), MATRICS Consensus Cognitive Battery (MCCB), and Quality of Life Scale (QLS) for community functioning. Treatment side effects were evaluated with the Simpson Angus Scale (SAS), Barnes Akathisia Rating Scale (BARS), Abnormal Involuntary Movement Scale (AIMS), UKU side effect rating scale (UKU), Liverpool University Neuroleptic Side Effect Rating Scale (LUNSERS), Yale-Brown Obsessive Compulsive Scale (YBOCS), and Barratt Impulsiveness Scale (BIS). Participants were reassessed on measures of psychosis and side effects weekly for the first four weeks, and every two weeks thereafter. The SANS and CGI were repeated at weeks 4 and 8, with the MCCB and QLS re-administered at week 8. Statistical analyses included the intent-to-treat sample (i.e., participants who received at least one dose of study medication) and consisted of linear mixed models for change in SANS total score (our primary outcome), and change in other clinical and side effects measures as a result of treatment.

**Results:**

Enrolled participants (eight male and two female) had a mean age of 37.2 years. Seven participants completed the study, with three participants dropping out. The mean final dose of levodopa for study completers was 835.7 mg (SD 170.1). Levodopa augmentation resulted in a significant improvement in negative symptoms, with a mean SANS reduction for study completers of 15.3 (SD 5.7). This equated to a mean SANS improvement of 25.5% (range 17% to 57%), with 43% of study completers experiencing > 20% improvement in SANS score. Notably, significant improvement in negative symptoms emerged after the first four weeks of treatment. There was no significant change in positive symptoms, nor other clinical outcomes or side effect measures.

**Discussion:**

Our findings suggest that levodopa augmentation of antipsychotics may be an effective and well-tolerated treatment for negative symptoms in schizophrenia. These findings, however, need replication in larger randomized controlled trials. With the dearth of available treatments for negative symptoms, levodopa augmentation may represent a novel pharmacologic strategy to address this critical unmet therapeutic need for schizophrenia.

